# New insights into the pathogenesis and therapeutics of episodic ataxia type 1

**DOI:** 10.3389/fncel.2015.00317

**Published:** 2015-08-19

**Authors:** Maria Cristina D’Adamo, Sonia Hasan, Luca Guglielmi, Ilenio Servettini, Marta Cenciarini, Luigi Catacuzzeno, Fabio Franciolini

**Affiliations:** ^1^Section of Physiology and Biochemistry, Department of Experimental Medicine, University of PerugiaPerugia, Italy; ^2^Department of Chemistry, Biology and Biotechnology, University of PerugiaPerugia, Italy

**Keywords:** episodic ataxia type 1, *KCNA1*, Kv1.1 potassium channel, point mutations, channelopathy, episodic ataxia type 1 therapy

## Abstract

Episodic ataxia type 1 (EA1) is a K^+^ *channelopathy* characterized by a broad spectrum of symptoms. Generally, patients may experience constant myokymia and dramatic episodes of spastic contractions of the skeletal muscles of the head, arms, and legs with loss of both motor coordination and balance. During attacks additional symptoms may be reported such as vertigo, blurred vision, diplopia, nausea, headache, diaphoresis, clumsiness, stiffening of the body, dysarthric speech, and difficulty in breathing. These episodes may be precipitated by anxiety, emotional stress, fatigue, startle response or sudden postural changes. Epilepsy is overrepresented in EA1. The disease is inherited in an autosomal dominant manner, and genetic analysis of several families has led to the discovery of a number of point mutations in the voltage-dependent K^+^ channel gene *KCNA1* (Kv1.1), on chromosome 12p13. To date *KCNA1* is the only gene known to be associated with EA1. Functional studies have shown that these mutations impair Kv1.1 channel function with variable effects on channel assembly, trafficking and biophysics. Despite the solid evidence obtained on the molecular mechanisms underlying EA1, how these cause dysfunctions within the central and peripheral nervous systems circuitries remains elusive. This review summarizes the main breakthrough findings in EA1, discusses the neurophysiological mechanisms underlying the disease, current therapies, future challenges and opens a window onto the role of Kv1.1 channels in central nervous system (CNS) and peripheral nervous system (PNS) functions.

## Introduction

Encoded by more than 70 genes, K^+^ channels make up the largest group of ion channels found in virtually all cells of the human body. A substantial number of diseases known as *channelopathies*, described in both humans and animals, result from either mutations or dysfunctions in K^+^ channels. Episodic ataxia type 1 (EA1) is one of the first *K*^+^ *channelopathies* that was identified and included in a continuously growing list (Adelman et al., 1995; Sicca et al., 2011; D’Adamo et al., 2013; Ambrosini et al., 2014; Guglielmi et al., 2015; Parolin Schnekenberg et al., 2015). In this report, we will review ours and others past work and achievements, and retrace the challenges encountered in pinning down the molecular and neurophysiological mechanisms underlying this neurological disorder. This undertaking will provide a comprehensive and up to date account of our present understanding of EA1, and indicate new directions for future studies.

## Clinical Findings

Episodic ataxia type 1 [EA1; OMIM 160120] is a neuromuscular disease described precisely by VanDyke et al. (1975). It is characterized by constant myokymia (fine twitching of groups of muscles, intermittent cramps and stiffness) and dramatic episodes of ataxia accompanied by spastic contractions of the skeletal muscles of the head, arms, and legs. In several cases patients show loss of motor coordination and balance. Attacks can last seconds or minutes, but in some cases many hours (Lee et al., 2004). Recently, episodes characterized by ataxia/dysarthria with concurrent hyperthermia (up to 40.3°C) that lasted for days were witnessed in-hospital (D’Adamo et al., 2015). The frequency of attacks is variable. Some patients present ataxia more than 15 times a day, while others less than once a month (VanDyke et al., 1975). Generally the onset of the disease is during childhood and can be triggered by traumatic physical or emotional events (Imbrici et al., 2008), as well as by stimuli like fever, startle response, abrupt movements, vestibular caloric stimulation, anxiety, repeat knee bends, exercise, ingestion of caffeine, and riding a *merry-go-round*. High temperatures may also precipitate attacks (Eunson et al., 2000).

Myokymia manifests during and between attacks, rarely in the absence of ataxia or other neurological deficits. In some EA1 patients myokymic activity is either absent on the electromyography (EMG; D’Adamo et al., 2015) or becomes apparent after the application of regional ischemia. Brunt and van Weerden (1990) proposed that the abnormal EMG responses originated peripherally. A recent study in patients harboring EA1 mutations reported alterations in axonal excitability parameters and proposed simple EMG protocols helpful to diagnose EA1 (Tomlinson et al., 2010).

Epilepsy is over-represented in EA1. Some affected patients can present tonic-clonic and partial seizures (Imbrici et al., 2008) and/or abnormal electroencephalograms (EEGs; VanDyke et al., 1975; Zuberi et al., 1999; Lee et al., 2004).

Since the first description of EA1, the phenotypic spectrum of the disease has widened considerably. Some affected individuals may also display: delayed motor development, choreoathetosis, carpal spasm, clenching of the fists, cognitive dysfunctions, expressive language delay and inability to learn a motor task (e.g., ride a bicycle; Zuberi et al., 1999; Demos et al., 2009). A short sleep phenotype has been recently reported (D’Adamo et al., 2015). Moderate muscle hypertrophy with generalized increase in muscle tone and bilateral calf hypertrophy was observed in some individuals. Others display apnoea, cyanosis, attacks of breathing difficulty (Zuberi et al., 1999; Shook et al., 2008), and skeletal deformities (Kinali et al., 2004; Klein et al., 2004). The magnetic resonance imaging (MRI) neuroimage is usually normal, albeit a family with cerebellar atrophy has been reported (Demos et al., 2009; Glaudemans et al., 2009). It must also be pointed out that phenotypic differences exist not only between families harboring different mutations, but also between individuals of the same family.

## Genetics

**Figure 1:**
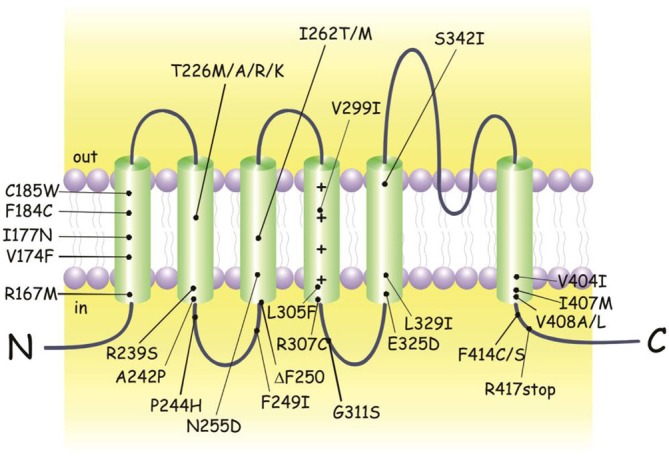
***KCNA1* mutations identified in episodic ataxia type 1 (EA1) individuals**. Cartoon showing the membrane topology of a human Kv1.1 subunit and the positions of the EA1 mutations. Up-dated and adapted from D’Adamo (2010).

EA1 is an autosomal dominant disorder involving heterozygous point mutations in the gene *KCNA1* on chromosome 12p13 which encodes for the voltage dependent K^+^ channel Kv1.1 (Figure 1; Browne et al., 1994, 1995; Litt et al., 1994; Comu et al., 1996; Imbrici et al., 2008). To date *KCNA1* is the only gene known to be associated with EA1. Since several individuals displaying typical EA1 symptoms do not carry variations in *KCNA1*, it is highly likely that defects in other genes may underlie the disease. In patients with a complex phenotypic presentation of EA1, the whole-exome sequencing (WES) can be a useful, time-saving, and cost-effective diagnostic tool (Tacik et al., 2015).

Most individuals diagnosed with EA1 have an affected parent, although de novo mutations have been identified (Demos et al., 2009; Lassche et al., 2014). Despite the identification of some nonsense and small deletion mutations, the variations in the genetic code responsible for EA1 are mainly missense point mutations (Figure 1; Eunson et al., 2000; Shook et al., 2008). The majority of individuals carrying a *KCNA1* variant express the EA1 phenotype. However, not all individuals with mutations phenotypically express EA1 making the penetrance of the disease incomplete. EA1 has a disease prevalence of approximately 1:500,000, albeit it may be an underestimation due to misdiagnosis or unreported cases.

The amino acid residues mutated in the Kv1.1 channel of EA1 patients are located at positions highly conserved throughout evolution, from Drosophila melanogaster to humans (Browne et al., 1994). Interestingly, four different mutations of the highly conserved threonine 226 located within the second transmembrane segment were discovered (Figure 1; Rajakulendran et al., 2007). Diverse phenotypic characteristics may result from virtually identical channel defects arising from similar mutations. For example epilepsy, infantile contractures, postural abnormalities, and skeletal deformities are expressed with the mutation p.Thr226Arg and not by p.Thr226Ala and p.Thr226Met mutations. Vast differences in the severity and frequency of attacks are observed even when the same EA1 mutation exists, as in identical twins (Graves et al., 2010). Indeed, while one twin sought treatment the other who was less severely affected did not require medication, suggesting that the symptom heterogeneity can also be attributed to a non-genetic factors (Graves et al., 2010; D’Adamo et al., 2015). Due to such a wide interfamilial and intrafamilial phenotypic variability genotype-phenotype correlations have been extremely difficult to establish (Kullmann, 2010). Nevertheless, recently some correlation attempts have been made (Graves et al., 2014).

## Molecular Pathogenesis

The functionality and characteristics of several mutant channels have been established by expression of the mutant gene in *Xenopus* oocytes and mammalian cell lines, thereby determining the molecular mechanisms underlying the EA1 mutations (Table 1; Adelman et al., 1995; D’Adamo et al., 1998, 1999; Zerr et al., 1998; Zuberi et al., 1999; Eunson et al., 2000; Manganas et al., 2001; Imbrici et al., 2003, 2006, 2007, 2008, 2009; Cusimano et al., 2004).

**Table 1:** **Summary of the main functional defects caused by EA1 mutations**.

Mutation	Main functional defects compared to wild-type channels	Reference
R167M	Not functional with dominant negative effect	Tomlinson et al. (2013)
V174F	Marked reduction of surface expression	Adelman et al. (1995)
I177N	Reduction of surface expression with dominant negative effect, positive shift of voltage dependence of activation, slower activation, faster deactivation	Imbrici et al. (2003)
F184C	Reduction of surface expression, positive shift of voltage dependence of activation, slower kinetic of activation	Adelman et al. (1995)
C185W	Not functional with dominant negative effect	Tomlinson et al. (2013) D’Adamo et al. (2015)
T226A/M	Marked reduction of surface expression, positive shift of voltage dependence of activation, slower deactivation, slower activation	Zerr et al. (1998)
T226R	Marked reduction of surface expression with dominant negative effect, positive shift of voltage dependence of activation, slower activation, slower deactivation	Zuberi et al. (1999)
T226K	Not functional with dominant negative effect	Chen et al. (2007)
R239S	Not functional with strong dominant negative effect	Adelman et al. (1995)
A242P	Marked reduction of surface expression, negative shift of voltage dependence of activation, slower activation, slower deactivation	Eunson et al. (2000)
P244H	No differences between wild-type and mutant	Eunson et al. (2000)
F249I	Marked reduction of surface expression, slower deactivation	Adelman et al. (1995)
ΔF250	N/A	Shook et al. (2008)
N255D	Not functional with dominant negative effect	Glaudemans et al. (2009)
I262M	Not functional with dominant negative effect	Lassche et al. (2014)
I262T	N/A	Klein et al. (2004)
V299I	Positive shift of voltage dependence of activation with dominant negative effect	Rajakulendran et al. (2009)
L305F	Dominant negative effect on the Kv1.1 potassium channel gating properties	Poujois et al. (2006)
R307C	Not functional with dominant negative effect, positive shift of voltage dependence of activation	Graves et al. (2010)
G311S	Reduction of surface expression, positive shift of voltage dependence of activation, faster C-type inactivation	Zerr et al. (1998)
E325D	Marked reduction of surface expression with strong dominant negative effect, 60mV positive shift of voltage dependence of activation, faster deactivation, faster activation, faster C-type inactivation	Adelman et al. (1995)
L329I	N/A	Knight et al. (2000)
S342I	N/A	Lee et al. (2004)
V404I	Small effect on surface expression, positive shift of voltage dependence of activation, slower activation, slower deactivation	Eunson et al. (2000)
I407M	Not functional with dominant negative effect	Tomlinson et al. (2013)
V408A	Faster activation and deactivation, faster C-type inactivation, faster recovery from inactivation	Adelman et al. (1995)
V408L	Faster C-type inactivation	Demos et al. (2009)
F414C	Not functional	Imbrici et al. (2008)
F414S	Not functional with dominant negative effect, positive shift of voltage dependence of activation	Graves et al. (2010)
R417stop	Not functional with dominant negative effect	Eunson et al. (2000)

By studying the properties of Kv1.1 channels bearing the EA1 point mutations, Adelman et al. (1995) first demonstrated that the mutant channels showed altered gating properties. In particular, they showed that V408A channels had faster activation-deactivation kinetics and C-type inactivation, while F184C channels displayed a 20 mV positive shift in voltage-dependence.

A heterozygote for a *KCNA1* mutation may or may not express the normal and mutant allele equally. The extent of functional impairment of a Kv1.1 channel is dependent on the type and number of mutated subunits that make up the tetrameric channel. As such, channels composed of two Kv1.1 wild-type and two mutated subunits present whole-cell and single channel properties intermediate between tetramers made up of four wild-type or four mutated subunits (D’Adamo et al., 1998). Co-expression of wild-type and mutant subunits for some EA1 mutations presented a dominant negative effect resulting in less than half the normal current, while for other mutations virtually no effect was observed (Zerr et al., 1998; D’Adamo et al., 1999, 2015; Rea et al., 2002; Imbrici et al., 2006, 2011; Graves et al., 2010). Overall, these studies demonstrated that allelic variations underlying EA1 exert variable *loss-of-function* effects on the outward K^+^ flux through the channel made-up of Kv1.1 subunits by altering channel expression and gating. However, in the nervous system Kv1.1, Kv1.2 and Kv1.4 subunits are the most amply expressed, and they often form heteromeric channels composed of Kv1.1/Kv1.2 and Kv1.1/Kv1.4 subunits. The question arose as to whether or not EA1 mutations could alter the properties of these channel types. In a seminal study, we were the first to demonstrate the co-assembly of human Kv1.2 and Kv1.1 subunits to form a novel channel with distinct gating properties markedly altered by EA1 mutations (D’Adamo et al., 1999). The deleterious effects of EA1 mutations on heteromeric channel function was also shown for Kv1.4–1.1/Kvβ1.1, a channel highly expressed in the hippocampus (Imbrici et al., 2006). Kv1.4 subunits confer fast N-type inactivation properties to Kv1.4–1.1 channels. Intriguingly, EA1 mutations which have normal surface expression reduce the rate of inactivation conferred by Kv1.4 by decreasing the affinity for the inactivation domain, while mutations which have reduced subunit surface expression increase the rate of N-type inactivation. The latter is due to a stoichiometric increase in the number of Kv1.4 subunits assembled in the tetramer (Imbrici et al., 2011). Thus, subunit surface expression may inversely affect distinct functional properties of the channel. In conclusion, these studies have clearly demonstrated that allelic variations in a single gene (*KCNA1*) also alter the function of other proteins that interact with Kv1.1. These mechanisms are expected to broaden the spectrum of the molecular defects caused by EA1 mutations.

Zn^2+^ ions that are released into the synaptic cleft modulate the activity of distinct members of ligand-gated and voltage-gated ion channels. Indeed, we have shown that channels composed of Kv1.1/Kv1.4/Kvβ1.1 subunits possesses both a high affinity (<10 μM) and a low affinity (<0.5 mM) site for Zn^2+^ ions. The intrinsic affinity of these channels for Zn^2+^ binding was shown to be enhanced by an EA1 mutation (e.g., F184C; Imbrici et al., 2007).

The precise contribution of Kv1.1 defects described here on signaling dysfunction, in the neural networks expressing this channel, is not clear. We will address this issue in the next paragraph.

## Neurophysiological Pathogenesis

The expression pattern of Kv1.1 channels within the CNS and PNS varies. Dysfunctions of circuits located in the cerebellum, hippocampus, cortex and PNS have been implicated in EA1. Here, we will attempt to correlate the anatomical and subcellular location of Kv1.1 channels with their physiological roles, molecular defects caused by EA1 mutations and clinical findings.

### Cerebellum

The cerebellum, known to play a crucial role in motor control and cognitive function, is implicated in EA1. In fact, EA1 is characterized by typical symptoms of cerebellar pathology. Kv1.1-containing channels show a discrete pattern of subcellular localization in the cerebellum. In particular, Kv1.1 and Kv1.2 are extensively expressed at Pinceau synapses formed between basket cell terminals and the initial segments of the Purkinje cell axons, and at the juxtaparanodal regions of virtually all the myelinated axons of the cerebellar white matter (McNamara et al., 1993; Wang et al., 1993, 1994; Laube et al., 1996; Rhodes et al., 1997). By contrast, Punkinje cells does not express Kv1.1 subunits (Khavandgar et al., 2005; Lorincz and Nusser, 2008).

In accordance with a prevalent expression of Kv1.1 subunits in basket cell terminals, the amplitude and frequency of inhibitory post-synaptic currents (IPSCs) in Purkinje cells, mediated by type A gamma-aminobutyric acid (GABA_A_) receptor activation, are increased by *in vitro* application of alfa dendrotoxin (α-DTX), a selective blocker of Kv1.1 and Kv1.2 channels (Southan and Robertson, 1998). We have previously proposed a model in which a reduction of delayed rectifier current passing through heteromeric channels composed of EA1 Kv1.2 and Kv1.1 mutated subunits increases excitability of presynaptic basket cell terminals, prolongs action potential duration and enhances Ca^2+^ ion influx. Larger amounts of GABA were predicted to be released from basket cell terminals which could inhibit action potential generation at the Purkinje axon hillock. As a consequence, the inhibitory output of the entire cerebellum to the rest of the brain could be markedly reduced, producing the disinhibition of deep cerebellar nuclei and cerebellar symptoms typically observed in EA1 patients (D’Adamo et al., 1999). By inserting the heterozygous p.Val408Ala mutation in one Kv1.1 allele a mouse model of EA1 that recapitulated the disease was generated. In particular, a procedure that mimics stress-fear responses induced motor dysfunctions in Kv1.1^V408A/+^ animals similar to EA1 (Herson et al., 2003). The p.Val408Ala is a homozygous lethal mutation that causes embryonic death between E3-E9. Conversely, heterozygous Kv1.1^V408A/+^ animals are viable. Electrophysiological recordings from cerebellar slices dissected from Kv1.1^V408A/+^ animals showed an increase in frequency and amplitude of spontaneous GABA_A_ IPSCs at cerebellar basket cell–Purkinje cell synapses compared to wild-type Kv1.1^+/+^ mice (Herson et al., 2003). The results of this study is in agreement with our postulated pathogenetic model of EA1 (D’Adamo et al., 1999). A more recent study provided further evidence supporting this model, namely basket cell terminals of Kv1.1^V408A/+^ animals had broader action potentials at half amplitude than in wild-type (Begum and Kullmann, 2011). Nevertheless, many questions concerning the abnormalities within cerebellar circuitries that underlie EA1 remain to be addressed. A basket cell axon forms branches which make synaptic contact with several Purkinje cells. Since Kv1.1 are highly concentrated at axonal branch points (Tsaur et al., 1992; Wang et al., 1994), this channel type may control the success rate of action potential propagation down the axon. Direct evidence showing how EA1 mutations alter this important process has not been provided to date. In addition, what determines the episodic nature of the cerebellar symptoms is unknown. A phenomenon akin to *spreading acidification of the cerebellar cortex* has been suggested (Chen et al., 1999, 2005; Ebner and Chen, 2003), although more conclusive data should be provided in this regard.

The motor performance of some EA1 patients is altered mostly during attacks when they display imbalance and uncontrolled movements, mostly triggered by emotional and/or physical stress. Kv1.1^V408A/+^ animals recapitulate the stress-induced motor dysfunctions often observed in patients. Indeed, pretreatment with low dose of isoproterenol, a β-adrenergic agonist, significantly decreased the latency to fall and increased the number of missteps of Kv1.1^V408A/+^ animals compared to WT littermates (Herson et al., 2003). However, in the resting condition or after exercise, the motor coordination of Kv1.1^V408A/+^ mice were normal.

### Hippocampus

The hippocampus of the limbic system is associated primarily with memory consolidation, and in particular with spatial memory. EA1 may be associated with cognitive symptoms and seizures and therefore, abnormalities within hippocampal circuitries have been suggested to play a role in this disease. Kv1.1, Kv1.2 and Kv1.4 are abundantly expressed in *Schaffer* collateral axons, as well as in the axons and terminals of the medial perforant pathway of the dentate gyrus. CA3 mossy fibre boutons that from *en passant* synapses with pyramidal neurons express a channel complex composed of Kv1.1, 1.4 and Kvβ1.1 known to regulate the activity-dependent spike broadening and glutamate release during high-frequency stimulation (Geiger and Jonas, 2000). EA1 mutations drastically alter the function of heteromeric channels composed of Kv1.1, Kv1.2, Kv1.4 and Kvβ1.1 subunits and may therefore, contribute to seizure susceptibility and cognitive symptoms in EA1 (Imbrici et al., 2006, 2011).

Kv1.1/Kv1.4/Kvβ1.1 channels possess high sensitivity to Zn^2+^ inhibition (*see above*). Although the effect of Zn^2+^ ions on brain excitability remains controversial, there are evidence that proof otherwise. For example, intracranial administration of Zn^2+^ salts has been associated with epileptiform activity and changes in Zn^2+^ modulation of GABA receptors have been implicated in the etiology of epilepsy. In addition, granule cell epileptiform activity is facilitated by the release of Zn^2+^ from recurrent mossy fibers (Timofeeva and Nadler, 2006). Mossy fibre boutons contain Zn^2+^ stored at high concentrations in pre-synaptic vesicles (approximately 300 μM; Frederickson, 1989). In the hippocampus, Zn^2+^ is released from mossy fiber terminals during synaptic activity (Assaf and Chung, 1984) into the synaptic cleft where they more than likely decrease the activity of channels composed of Kv1.1/Kv1.4/Kvβ1.1 subunits. Imbrici et al. (2007) suggested that Zn^2+^ inhibition of Kv1.1-containing channels, combined with the intrinsic loss of channel function, caused by the *KCNA1* mutations, would exacerbate EA1 symptoms. These combined inhibitory effects on K^+^ currents would impair the integration and transmission of signals within distinct brain areas such as the hippocampus and cerebellum (Imbrici et al., 2007). Intriguingly, a distinct EA1 mutation (F184C) increased several folds the intrinsic Zn^2+^ sensitivity of Kv1.1-containing channels and epilepsy was reported in the patient carrying this mutation (Browne et al., 1994; Cusimano et al., 2004). Investigations aimed at establishing whether Zn^2+^ plays a role in cognitive, epileptic and cerebellar EA1 symptoms are ongoing in our laboratory. The neurophysiological role of Kv1.1 channels in the hippocampus, and the relevant mechanisms underlying seizure susceptibility has been investigated in animal models of Kv1.1 *channelopathies.* We have recently reviewed the progress in the neurobiology of “*Kv1.1 channelepsy*” in the following publications: D’Adamo et al. (2013, 2014) that are available for further insights.

### Peripheral Nervous System

In the myelinated axons of the PNS it is possible to recognize several specialized domains: the node of Ranvier, paranode, juxtaparanode and internode. Kv1.1, Kv1.2 and the accessory subunits Kvβ1.2 form a macromolecular complex in the juxtaparanodal region and in axon branching points but is absent at both the end-plate and muscle fibers (Tsaur et al., 1992; Wang et al., 1993, 1994; Poliak et al., 1999; Vacher et al., 2008). Neuromytonia and myokymia are present in the limbs and particularly in the face and hands of EA1 patients. The EMG of myokymic patients display patterns of either rhythmic or arrhythmic singlets, duplets, or multiplets that may be apparent after application of regional ischemia (*e.g.*, using an inflated sphygmomanometer cuff applied around the upper or lower arm for up to 15 min). Patients manifesting neuromyotonia or myokymia, and harboring different *KCNA1* mutations displayed 100% higher axonal hyperexcitability compared to healthy controls (Tomlinson et al., 2010).

The neurophysiological role of Kv1.1 channels in neuromuscular transmission has been elucidated by using transgenic mouse models. These studies pointed out that juxtaparanodal Kv1.1 channels are critical regulators of axonal excitability. In fact, Kv1.1 ablation in mice causes repetitive neuronal activity in the phrenic nerve resulting from both spontaneous and stimulus-evoked nerve-backfiring at pre-terminal axon transition zones. At these zones axons change from myelinated to non-myelinated (Smart et al., 1998; Zhou et al., 1998, 1999; Vabnick et al., 1999). This scenario is consistent with computer simulations showing that lack of juxtaparanodal Kv1.1 channels lead to reentrant excitation of nodes due to nerve backfiring at axon transition zones of myelinated nerve terminals (Zhou et al., 1999).

Slower repolarization phases of the compound action potentials and repetitive electrical activity was observed in the phrenic nerve-muscle preparation of Kv1.1^−/−^ mice (Smart et al., 1998; Zhou et al., 1998, 1999; Vabnick et al., 1999). These findings let us postulate that the respiratory symptoms reported in some individuals with EA1 may also be caused by abnormal transmission of signals along the phrenic nerve (Shook et al., 2008). By using *lateral gastrocnemius* nerve–muscle preparations, *in vivo* and *in vitro*, we observed that Kv1.1^V408A/+^ mice displayed spontaneous *myokymic*—like discharges (repeated singlets, duplets or multiplets) and abnormal Ca^2+^ signals (Brunetti et al., 2012). Single nerve stimulation or stresses, such as fatigue, lower temperatures and ischemia, exacerbated these electrical abnormalities. Brunt and van Weerden (1990) observed an increase in spontaneous activity and burst frequency in EA1 individuals during the first few minutes of limb ischemia induced by an inflated sphygmomanometer. In some instances, after 5–10 min of ischemia the spontaneous discharges disappeared. In contrast, recruitment of new and large multiplets and enlargement of pre-existing complexes with extra spikes were also observed following ischemia. This excess of activity began 0.5–1 min after reversal of ischemia, reached a maximum at 2–5 min and gradually declined over 10–15 min. Strikingly, Kv1.1^V408A/+^ mice recapitulated faithfully also these EA1 neuromuscular findings (Brunetti et al., 2012). Cooling-induced severe neuromyotonia and ataxia were also observed in a rat model of EA1 (Ishida et al., 2012). The distinct expression of Kv1.1 in axonal structures and the fact that spontaneous discharges are present, *in vivo*, despite severing the motor nerve supplying the Kv1.1^V408A/+^ muscle or, in *in vitro* investigations, indicate that the sciatic nerve is an intrinsic site of origin of anomalous re-excitation. This represents a more clear indication that neuromyotonia/myokymic activity in EA1 patients originates also from hyper-excitability of the peripheral nerve endings.

## Ion Channel-Modulating Drugs in EA1 Therapy

Several drugs improve symptoms in patients with EA1 but so far, with the lack of studies and trials comparing efficacy of these drugs, no single medication has been proven effective.

* Acetazolamide* (ACTZ), a carbonic anhydrase inhibitor, is used to reduce frequency and severity of EA1 attacks. While some EA1 patients experience improvement with ACTZ, responsiveness to ACTZ treatment is only occasional (Jen et al., 2007). The mechanism by which ACTZ reduces symptoms in responsive EA1 patients is unclear. Interestingly, stress-induced motor dysfunctions in animals with a EA1 mutation (Kv1.1^V408A/+^) are ameliorated by ACTZ, thus supporting its therapeutic potential (Herson et al., 2003). ACTZ may act by modulating intracellular pH or HCO_3_^−^ gradient. It affects CSF pH that may be responsible for its therapeutic effect, since patients with ACTZ-responsive ataxia have regional alkalosis. Normalization of intracellular pH after ACTZ treatment has been observed in episodic ataxia type 2 (EA2) and cerebellar ataxic patients (Bain et al., 1992; Sappey-Marinier et al., 1999). As a consequence of pH adjustments, channels and ionic conductance across neuronal membranes may be modulated, causing membrane hyperpolarization and a decrease in excitability that may manifest as the observed reduction in attacks. On the other hand, ACTZ’s effect may be due to the development of a HCO_3_^−^ gradient that opposes the depolarizing Cl^−^ shift responsible for the inversion of the signal, from an inhibitory to excitatory, mediated by the ligand-gated ion channel GABA_A_. Alternatively, ACTZ may reduce excitability of GABAergic interneurons as a consequence of intracellular alkalization (Herson et al., 2003). However, ACTZ possess a potent ability to open calcium-activated potassium channels (BK) and this action was proposed as a possible therapeutic mechanism improving myotonia (Tricarico et al., 2013). Unfortunately, the decrease in efficacy of ACTZ over time (Zuberi et al., 1999) and the development of adverse side effects (Lubbers et al., 1995) has led to the discontinuation of ACTZ treatment in many of those who are ACTZ-responsive. Long-term side effects of ACTZ include nephrolithiasis, hyperhidrosis, paresthesias, rash, diffuse weakness, and gastrointestinal discomfort (Kotagal, 2012). ACTZ treatment should be avoided in individuals with liver, renal, or adrenal insufficiency.

The antiepileptic drug, *sulthiame* is another carbonic anhydrase inhibitor that reduces frequency of attacks. During treatment with sulthiame, however, abortive attacks lasting a few seconds were still noticed. Paresthesias and intermitted carpal spasms were the troublesome side effects observed in patients treated with this drug.

*Phenytoin* is a modulator of voltage-gated Na^+^ channels, currently used as antiepileptic drug, that is also capable of decreasing ataxia (VanDyke et al., 1975) and myokymia (Gancher and Nutt, 1986) in EA1 patients. Phenytoin controlled seizures by blocking sustained high frequency repetitive action potentials (VanDyke et al., 1975). Moreover, it improved muscle stiffness and motor performance (Kinali et al., 2004). In other reported cases, however, phenytoin showed no effect (Brunt and van Weerden, 1990; Zuberi et al., 1999). Despite its possible therapeutic potential, phenytoin should be used with caution particularly in young individuals, as it may cause permanent cerebellar dysfunction and atrophy (De Marco et al., 2003).

*Carbamazepine* (CBZ) stabilizes the inactivated state of voltage-gated Na^+^ channels, making fewer of these channels available to subsequent opening. Also CBZ has been used to ameliorate EA1 symptoms (Eunson et al., 2000). In an animal model of temporal lobe epilepsy (Kv1.1^S309T/+^ rats), CBZ administration ameliorated behavioral phenotypes and abnormal discharges occurring in the cortex and hippocampus (Ishida et al., 2012). CBZ significantly reduced the frequency of attacks in members of a large Australian family (Hand et al., 2001). Similarly, all 16 affected members of a large British family with V404I mutation, who displayed typical EA1 attacks, responded well to CBZ (Eunson et al., 2000). In some cases, however, the CBZ initial response was not sustained (Zuberi et al., 1999; Eunson et al., 2000).

Responses of EA1 patients to medication are variable, being observable also amongst members of families harboring the same *KCNA1* mutation. Indeed Scheffer et al. (1998) reported patients from a family harboring a V404I mutation who were not responsive to treatment with ACTZ, while in another family with the same mutation a 50% reduction in attacks after ACTZ treatment was observed (Tacik et al., 2015).

## Concluding Remarks

Fourty years ago, VanDyke et al. (1975) described EA1, distinctively. Since then, a wealth of new findings, resulting from genetics and electrophysiological investigations, clarified the causes underlying this neurological disease, which emerges as severe in some individuals. Concerning this last issue, it is still unclear why patients, carrying even the same *KCNA1* mutation, exhibit very different disease phenotypes and severity. Elusive epigenetic factors are often invoked to explain such remarkable differences. Unclear are also the mechanisms determining the episodic nature of EA1 and how physiological stresses, which stimulate otherwise normal responses in healthy subjects, trigger attacks of ataxic gait in patients. Finally, the lack of a highly specific drug able, ideally, to enhance the activity of Kv1.1-containing channels and offset the biochemical and biophysical defects caused by the *KCNA1* mutations, makes treatment of EA1 problematic. The major challenges in the near future should be aimed at finding answers to these and other puzzling issues relevant to fully understand EA1.

## Conflict of Interest Statement

The authors declare that the research was conducted in the absence of any commercial or financial relationships that could be construed as a potential conflict of interest.

## Abbreviations

ACTZ: Acetazolamide
α-DTX: Alfa Dendrotoxin
CBZ: Carbamazepine
CNS: Central Nervous System
EA1: Episodic Ataxia type 1
EA2: Episodic Ataxia type 2
EEG: Electroencephalography
EMG: Electromyography
GABA: Gamma-Aminobutyric Acid
IPSCs: Inhibitory Post-Synaptic Currents
MRI: Magnetic Resonance Imaging
PNS: Periferal Nervous System
WES: Whole-exome sequencing.
